# Elafibranor Inhibits Chronic Kidney Disease Progression in NASH Mice

**DOI:** 10.1155/2019/6740616

**Published:** 2019-06-19

**Authors:** Hung-Cheng Tsai, Fu-Pang Chang, Tzu-Hao Li, Chih-Wei Liu, Chia-Chang Huang, Shiang-Fen Huang, Ying-Ying Yang, Kuei-Chuan Lee, Yun-Cheng Hsieh, Ying-Wen Wang, Tzung-Yan Lee, Yi-Hsiang Huang, Ming-Chih Hou, Han-Chieh Lin

**Affiliations:** ^1^Department of Medicine, Taipei Veterans General Hospital, Taipei, Taiwan; ^2^Department of Medicine, National Yang-Ming University School of Medicine, Taipei, Taiwan; ^3^Department of Pathology, Taipei Veterans General Hospital, Taipei, Taiwan; ^4^Division of Allergy and Immunology, Taipei Veterans General Hospital, Taipei, Taiwan; ^5^Institute of Clinical Medicine, National Yang-Ming University School of Medicine, Taipei, Taiwan; ^6^Chia-Yi Branch of Taichung Veterans General Hospital, Chiayi, Taiwan; ^7^Division of Infection, Taipei Veterans General Hospital, Taipei, Taiwan; ^8^Division of Gastroenterology and Hepatology, Taipei Veterans General Hospital, Taipei, Taiwan; ^9^Division of General Medicine, Taipei Veterans General Hospital, Taipei, Taiwan; ^10^Graduate Institute of Traditional Chinese Medicine, Chang Guang Menorial Hospital, Linkou, Taiwan

## Abstract

Identification of new pharmacological approaches to inhibit the excessive fat intake-induced steatohepatitis and chronic kidney disease (CKD) is important. High-fat diet (HFD)-induced steatohepatitis and CKD share common pathogenesis involving peroxisome proliferator-activated receptor (PPAR)-*α* and -*δ*. Elafibranor, a dual PPAR*α*/*δ* agonist, can ameliorate the HFD-induced steatohepatitis. Nonetheless, the effects of HFD-induced CKD had not yet explored. This study investigated the effects of elafibranor (elaf) on the progression of HFD-induced CKD in mice.* In vivo* and* in vitro* renal effects were evaluated in HFD-elaf mice receiving 12 weeks of elafibranor (from 13^th^ to 24^th^ week of HFD feeding) treatment. In elafibranor-treated HFD mice, increased insulin sensitivity, reduced obesity and body fat mass, decreased severity of steatohepatitis, increased renal expression of PPAR*α*, PPAR*δ*, SIRT1, and autophagy (Beclin-1 and LC3-II) as well as glomerular/renal tubular barrier markers [synaptopodin (podocyte marker), zona occludin-1, and cubulin], reduced renal oxidative stress and caspase-3, and less urinary 8-isoprostanes excretion were observed. Aforementioned benefits of elafibranor were associated with low renal tubular injury and tubulointerstitial fibrosis scores, less albuminuria, low urinary albumin-to-creatinine ratio, and preserved glomerular filtration rate. Acute incubation of podocytes and HK-2 cells with elafibranor or recombinant SIRT1 reversed the HFD-sera-induced oxidative stress, autophagy dysfunction, cell apoptosis, barrier marker loss, albumin endocytosis, and reuptake reduction. Besides hepatoprotective and metabolic beneficial effects, current study showed that elafibranor inhibited the progression of HFD-induced CKD through activation of renal PPAR*α*, PPAR*δ*, SIRT1, autophagy, reduction of oxidative stress, and apoptosis in mice with steatohepatitis.

## 1. Introduction

High-fat diet (HFD) intake and obesity have been associated with onset and progression of steatohepatitis and chronic kidney disease (CKD) [1–4]. In obesity, hemodynamic and morphological changes, together with other factors such as systemic inflammation, oxidative stress, and metabolic dyshomeostasis, may result in steatohepatitis and CKD and ultimately lead to cirrhosis and ESRD. Obesity was the second most highly predictive factor to predict end-stage renal disease (ESRD), even independent of diabetes and hypertension [4, 5].

Obesity-related CKD has been characterized by proteinuria, inflammation, and fibrosis [6]. Steatohepatitis and CKD share common pathogenic factors, incidences of both of them increased in severe obese patients [1–3, 5]. HFD-fed mice is a widely used experimental model to induce obesity, CKD, and steatohepatitis [7].

The perioxisome proliferator-activated receptors (PPAR)-*α* and PPAR*δ* are crucial for the regulation of inflammation, oxidative stress, and metabolic dyshomeostasis in obese individuals with steatohepatitis [8, 9]. Elafibranor is a novel dual PPAR*α*/*δ* agonist to reduce impaired metabolism, inflammation, and fibrosis in obese patients and animals with steatohepatitis [10, 11].

PPAR*α* agonists attenuate albuminuria and renal fibrosis in diabetic animals [12]. In diabetes mice, downregulated renal PPAR*δ* expressions result in heavy albuminuria, renal inflammation, and fibrosis [13, 14]. The advantage of dual PPAR*α*/*δ* agonist with respect to renal function was demonstrated by a lower risk of serum creatinine elevation with elafibranor (PPAR*α*/*δ* agonist) user than fenofibrate (PPAR*α* agonist, 7.1% versus 17.2%) user with obesity and steatohepatitis [12]. Sirtuin 1 (SIRT1) has been reported to have proautophagic, antioxidative stress, anti-inflammation, and antiapoptotic effects [14–19]. Both PPAR*α* and PPAR*δ* activation can stimulate SIRT1 expression [20, 21]. Hepatic and renal SIRT1 expressions are downregulated in high fat diet-induced obese animals with steatohepatitis and CKD [14, 15]. Renal SIRT1 activation attenuates diabetic albuminuria and ameliorates renal fibrosis [16, 17]. Pharmacologic activation of SIRT1 can alleviate steatohepatitis and CKD in obese animals [18, 19].

Accordingly, this study evaluated the PPAR*α*/*δ*-activated SIRT1-mediated molecular mechanism and effects of chronic elafibranor treatment on the progression of CKD in HFD-fed obese mice with steatohepatitis.

## 2. Materials and Methods

Additional information was included in the* supplementary materials and methods *(available here)

### 2.1. Animals

Male C57BL/6 mice (Jackson Laboratories, Bar Harbor, ME), 8 weeks old, were housed in temperature- and humidity-controlled rooms, kept on 12 h light/dark cycle, and provided unrestricted amounts of food and water. This study was approved by the Animal Experiments Committee of Yang-Ming University. Mice were provided with normal chow (NC, Laboratory Autoclavable Rodent Diet 5010) as NC group or a high-fat-diet (HFD, 60% kcal in fat) as HFD groups. In concordance with previous reports [20, 21], CKD including albuminuria (renal damage) and decreased creatinine clearance (poor renal function) were observed in HFD-24w mice [mice feeding with 24 weeks of HFD] in preliminary experiments (n=3).

**Figure 1:**
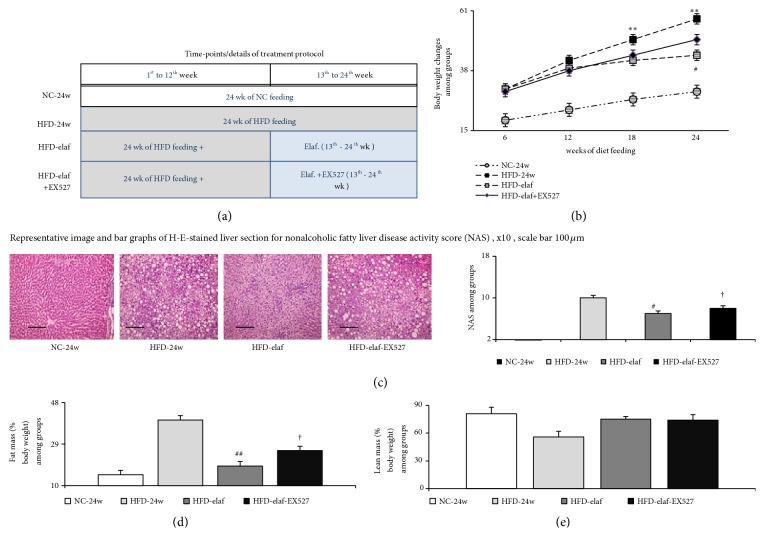
*Chronic dual PPARα/δ agonist, elafibranor, treatment improved severity of adiposity in HFD mice with steatohepatitis*. (a) Treatment protocol; (b) growth curves of HFD or NC mice: elafibranor decreased the trend of increased body weight, which can be abolished by concomitant SIRT1 inhibitor (EX527) treatment. Food and water consumption was estimated by daily observation at the time of feeding, and body weight was recorded every 2 weeks. (c) Representative image and bar graphs of H-E stain for hepatic steatosis: elafibranor decreased the severity of steatohepatitis; (d-e) whole body fat and lean mass of mice: elafibranor significantly reduced the fat mass of HFD mice. *∗*, *∗∗p*<0.05,0.01 versus NC-group. ^#^,^##^p<0.05, 0.01 versus HFD-group; †p<0.05 versus HFD*-*elaf group.

The groups (Figure 1(a)) of C57BL/6 mice included the following:* NC-24w* group (n=5)/*HFD-24w* group (n=8) continuously fed NC/HFD for 24 weeks were administered vehicle for 12 weeks from 13^th^ to 24^th^ week of NC/HFD feeding;* HFD-elaf* group (n=8) continuously fed HFD for 24 weeks were administered by oral gavage elafibranor (3mg/kg/day) for 12 weeks from 13^th^ to 24^th^ week of HFD feeding, at which time steatohepatitis, albuminuria, and a decrease in GFR developed. This dose of elafibranor (3mg/kg/day) has been demonstrated previously to decrease the progression of steatohepatitis [10, 12].

### 2.2. Blood Pressure, Metabolic Demands, and Renal Function

The mouse was placed in a metabolic cage and had free access to diet and water. Then, 24-hour urine sample was collected at 3 consecutive days, and the average of 3-day urine was calculated. The supernatant of centrifuged 3-day urine samples was used for various analyses, including albumin, creatinine, and 8-isoprostane [marker of oxidative stress]. Urinary albumin-to-creatinine ratio (ACR) was calculated as ACR = urine albumin/urine creatinine (*μ*g/mg). One day later, mouse GFR was measured after a single injection of FITC-inulin. The GFR was calculated using a two-compartment model of two-phase exponential decay. All above measurements were undergone in Taiwan Mouse Clinic (National Phenotyping and Drug Testing Center) on week 24 of the feeding regimen for HFD-24w/HFD-elaf/NC-24w groups.

Mouse GFR was measured by single injection of FITC-inulin clearance as described previously, modified to minimize plasma volume. The GFR was calculated using a two-compartment model of two-phase exponential decay. Briefly, dialyzed FITC-inulin (3.74 *μ*l/g body wt, Sigma-Aldrich, Inc., St. Louis, MO) was injected retro-orbitally under light anesthesia induced using isoflurane (Baxter Pharmaceutical Products, Deerfield, IL). The anesthesia lasted ~20s. Approximately 20*μ*l of blood was collected via the saphenous vein at 10, 35, 55, and 75 min after injection of FITC-inulin for the determination of FITC concentration.

### 2.3. Basal Measurements

Glucose tolerance test (GTT) was performed after overnight (16 h) fasting by intraperitoneal injection of D-glucose (2 mg/g body weight, Sigma-Aldrich, Inc., St. Louis, MO). Blood glucose was measured at 0, 30, 90, and 120 minutes using blood obtained by tail nicking using a One Touch glucometer (One Touch Ultra2, Life Scan, Johson&Johson, USA). All animals continued their initial feeding regimen until scarification. Under anesthesia, 2 days after stabilization and overnight fasting, heparinized-blood (from the inferior-vena-cava, abdominal-aorta, and heart-chamber) and the liver/kidney were collected and weighted.

### 2.4. Serum and Tissue Metabolic and Inflammatory Profiles

Serum biochemistry data, triglyceride, insulin, TNF*α*, and IL-6, caspase-3/7 activity as well as renal IL-6/TNF-*α* levels, myeloperoxidase (MPO) and SIRT1/caspase 3 activities, and hepatic SIRT1 activity were measured.

### 2.5. Histologic Analysis

Nonalcoholic fatty liver disease activity score (NAS) was measured by H-E-stained liver section. The H-E and periodic acid-Schiff (PAS)-stained renal section was evaluated to score the renal tubular damage and tubulointerstitial fibrosis. With an ApopTag Peroxidase* In Situ* Apoptosis Detection Kit (Chemicon, CA, USA), glomerular and tubules cells undergoing apoptosis were calculated.

### 2.6. Renal Electron Microscopic and Immunofluorescence Analysis

Then, the isolated membranes and autophagosomes on the ultrathin section in the proximal renal tubule of kidney were calculated by electron microscopy at 1,200x magnification. Meanwhile, each slide was evaluated for the numbers of cubulin/synaptopodin (+) cells per 1mm^2^ in the FITC images.

### 2.7. Protein and mRNA Measurements

Mouse podocytes and HK-2 cells were purchased from the CELPROGEN (3914 Del, Amo Blvd, Suite 901, Torrance, CA 90503) and Bioresource Collection and Research Center (BCRC, Hsin-Chu, Taiwan). Then, various proteins and mRNAs (with primers listed in Table 1) were measured in podocytes/HK-2 cell lysates, glomerular and tubular fractions of renal homogenates.

### 2.8. Roles of SIRT1-Autophagy on Elafibranor-Related Effects on HFD-Sera-Pretreated Podocytes and HK-2 Cells

HFD/NC-sera were obtained from NC-24w and HFD-24w mice. To mimic the impacts of circulating factors of HFD mice on abnormalities of renal microenvironment, various measurements were undertaken in 10% HFD-sera-pretreated podocytes/HK-2 cells. Significantly, 10% HFD-sera incubation suppressed the SIRT1 activity in cell lysates of podocytes/HK-2 cells. A preliminary dose-finding experiment revealed that, among different concentrations (5, 10, 15, and 30*μ*M) of elafibranor, maximal stimulation of SIRT1 activity on HFD-sera-pretreated cells was noted at 15*μ*M of elafibranor. Meanwhile, siSIRT1 was transfected into cells and maximal blockade of elafibranor-activated SIRT1 activity was noted at 100*μ*M.

Meanwhile, we found that rSIRT1 (300*μ*M) had similar effects as elafibranor (15*μ*M) to reverse HFD-sera-suppressed SIRT1 activity in cells. To examine the SIRT1-mediated effects of elafibranor on autophagy, HFD-sera-pretreated cells were incubated with bafilomycin A1 (BAF, 100ng/mL, a blocker of autophage flux) concomitantly with elafibranor (15*μ*M) or rSIRT1 (300*μ*M). For the following* in vitro* experiments, vehicle (V), elaf, elaf+siSIRT1, rSIRT1, rSIRT1+BAF group in either HFD-sera- or NC-sera-pretreated cells were included.

### 2.9. Albumin Endocytosis or Albumin Reuptake of HFD-Sera-Pretreated Podocytes and HK-2 Cells

For albumin endocytosis and albumin reuptake experiments, pretreated podocytes/HK-2 (1x10^5^ cells) cells were incubated with 1.5 mg/ml human FITC-albumin (MP Biomedicals, Santa Ana, CA) in Ringer solution at either 4°C or 37°C for 1 hour. Afterward, for spectrofluorometric measurements, podocytes/HK-2 were lysed in 20 mM MOPS with 0.1% Triton X-100. FITC-fluorescence was measured using an excitation wavelength of 490 nm and an emission wavelength of 540 nm by a fluorescence plate reader (Synergy HT; Biotek Instruments, Winooski, VT). The amount of protein in the lysates was measured using the bicinchoninic acid (BCA) assay (Pierce, Rockford, IL) and the amount of cell associated FITC-albumin was expressed as FITC-albumin (mg/mL) to compare degree of albumin endocytosis/reuptake between groups.

### 2.10. SIRT1-Autophagy Protein and mRNA Levels in Cultured Podocyte and HK-2 Cells

Notably,same protocol in albumin endocytosis (podocyte) and reuptake (HK-2) experiments was used to prepare cells for this part. After supernatants were collected for 8-isoprostane, caspase 3/7 activity was measured by the ELISA and luminescent substrate assay (Caspase-Glo assay; promega). Proteins and mRNAs were extracted, and cell lysates were used for SIRT1 activities measurement by Biomol SIRT1 fluorescence assay kit (AK-555; Biomol, Farmingdale, NY, USA).

Specifically, for calculation of autophagy flux index, cells were treated with 125nM of Bafilomycin A1 [inhibitors for maturation step of autophagosome including lysosomal enzyme activity and fusion of autophagosomes with lysosomes], in DMSO 2 hours before harvest to obtain cell lysates for measurement of LC3-II protein expression. Then, autophagy flux index was calculated by the formula [autophagy flux index=LC3-II (indicator of autophagosomes formation) expression levels with Bafilomycin A1 (100ng/mL)/LC3-II expression levels without Bafilomycin-A1]. LC3-II expression was normalized by its GADPH expression level.

### 2.11. Apoptotic and Barrier Markers (+) Podocytes and HK-2 Cells

Cells were stained with Annexin V and 7-amino-actinomycin D (7-AAD; BioLegend, San Diego, CA) and analyzed by flow cytometry with FCSCanto II (BD Biosciences, Mississauga, ON). For immunofluorescence (IF) staining, cells were fixed in paraformaldehyde followed by permeabilization with 0.025% digitonin in PBS. After washing, the cells were subsequently incubated at RT with synaptopodin/cubulin/LC3-II antibodies, FITC-conjugated secondary antibody. After washing with PBS, optical section data for % of synaptopodin/cubulin/LC3-II (+) area on each slide were evaluated.

### 2.12. Statistical Analysis

Results are presented as means±SD. Data were analyzed by ANOVA and Student-Newman-Keuls tests for multiple comparisons or by Student's *t*-test for unpaired data between two groups. Statistical significance was accepted at the* P* < 0.05 level.

## 3. Results

### 3.1. Chronic Elafibranor Treatments Improve Metabolic Profiles in HFD Mice

**Figure 2:**
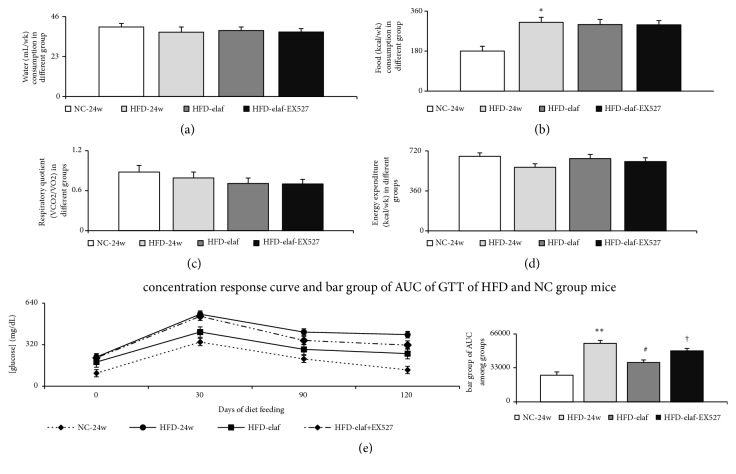
*Chronic elafibranor treatments increased insulin sensitivity of HFD mice with steatohepatitis*. Elafibranor had no effects on water ((a), mL/wk) and food ((b), kcal/wk) consumption; parameters of metabolic demands, respiratory quotient (c)/energy expenditure (d); (e) concentration-response curve and area under curve (AUC) of glucose tolerance test (GTT): elafibranor significantly improved the GTT, which was abolished by concomitant SIRT1 inhibitor (EX527) treatment. *∗*, *∗∗p*<0.05,0.01 versus NC-group; #p<0.05 versus HFD-group; †p<0.05 versus HFD-elaf group.

In comparison with NC-24w group, 24 weeks of HFD feeding induced hyperglycemia, hyperinsulinemia, abnormal GTT, higher homeostasis model assessment-insulin-resistance (HOMA-IR) index, more food consumption, greater incremental trend of body weight, higher serum/hepatic triglyceride level, and higher whole body fat mass were observed in HFD-24w group (Figures 1, 2(b), and 2(e), Table 2). Nonetheless, water consumption and metabolic demands [respiratory quotient (average whole body CO_2_ production/O_2_ consumption) and energy expenditure] were not different between NC group and HFD-group (Figures 2(a), 2(c), and 2(d)).

In HFD group, the beneficial effects of elafibranor with respect to hepatic steatosis, whole body fat mass, and GTT were counteracted by EX527 (a specific SIRT1 inhibitor), but not in body weight changes (Figures 1(b)–1(d) and 2(e), Table 2).,

### 3.2. Characteristics of CKD in HFD Mice with Steatohepatitis

**Figure 3:**
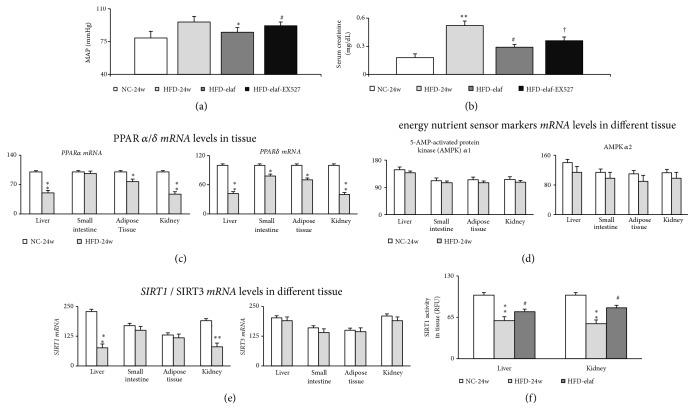
*Chronic elafibranor treatments improved the renal function through the activation of renal SIRT1 in HFD mice with steatohepatitis and CKD*. (a) Mean arterial blood pressure (MAP, mmHg); (b) serum creatinine (mg/dL); (c) PPAR *α*/*δ*; and (d-e) energy nutrient sensor markers* mRNA* levels (%/18S) in main target tissue of HFD mice; (f) liver and renal SIRT1 activity (RFU, relative fluorescence units). *∗*, *∗∗p*<0.05,0.01 versus NC-group; #p<0.05, 0.01 versus HFD-group; †p<0.05 versus HFD-elaf group.

**Figure 4:**
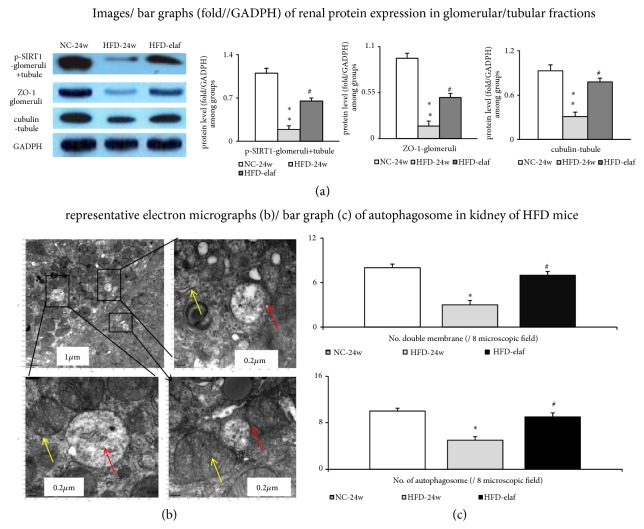
*Increased SIRT1 expression was accompanied by restoration of renal glomerular and tubular barrier protein expression in elafibranor-treated HFD mice with CKD*. (a) Representative image/bar graph of various protein expression in glomeruli/tubular fraction. Representative electron microscopy (EM) images (1,200x) (b)/bar graphs (c) of average number of double membrane (yellow arrow) and autophagosome (red arrow) in eight randomly selected fields in the proximal renal tubule of kidney of HFD mice, decrease in the number of double membrane and autophagosome was restored by elafibranor treatment. *∗*, *∗∗p*<0.05,0.01; ^#^p<0.05 versus HFD-group.

**Figure 5:**
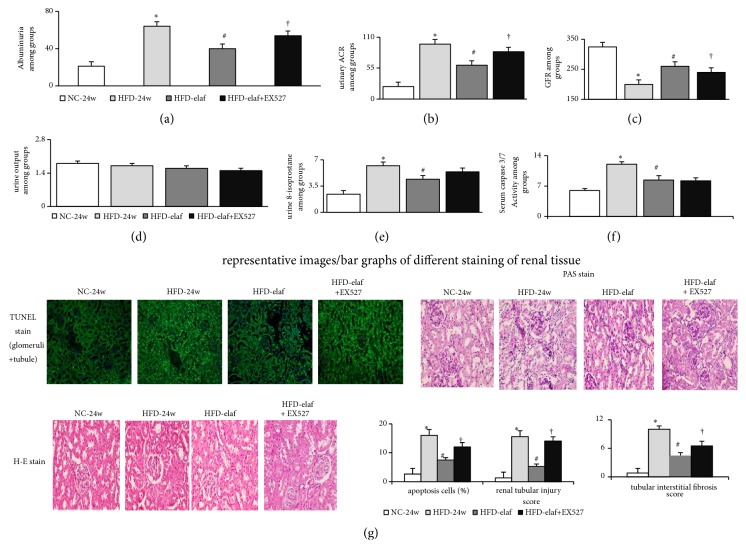
*Chronic elafibranor treatment suppressed the progression of CKD through the inhibition of renal oxidative stress/apoptosis in HFD mice with steatohepatitis*. (a) Albuminuria (urine albumin, *µ*g/day); (b) urinary albumin-to-creatinine ratio (ACR, albumin *µ*g/mg of creatinine); (c) glomerular filtration rate (GFR, *µ*L/min); (d) urine output; (e) urine 8-isoprostane (ng/mg cr) secretion; (f) serum caspase 3/7 activity; (g) histologic images/bar graphs of severity of renal injury. Renal tubular damage was assessed using a tubular damage score including atrophy and flattening of proximal tubule epithelial cells, and tubular dilation: 0 = normal; 1 = <20%; 2 = 20 to 40%; 3 = 40 to 60%; 4 = 60 to 80%; and 5 = 80%. The features of tubulointerstitial fibrosis include tubular atrophy/dilatation, presence of mononuclear inflammatory cells, widening of interstitial spaces with deposition of extracellular matrix, interstitial cell proliferation and wrinkling or thickened tubular basement membrane. *∗p*<0.05 versus NC-group; #p<0.05, 0.01 versus HFD-group; †p<0.05 versus HFD-elaf group.

The CKD findings in HFD mice include increased serum creatinine, increased albuminuria, urine ACR, decreased GFR, decreased renal PPAR*α*/*δ*, and SIRT1 expressions/activity (Figures 3(b), 3(c), 4(a), and 5(a)–5(c)).

Although no difference in water consumption and urine output was observed, significantly, increased kidney weight, renal MPO activity, and urinary 8-isoprostanes excretion were observed in HFD-group with CKD compared with that in the NC group (Table 2, Figures 2(a), 5(d), and 5(e)).

### 3.3. Elafibranor Normalizes Hepatic and Renal SIRT1 Expression in HFD Mice with Steatohepatitis and CKD

In the HFD group, a general reduction in liver, adipose tissue, and renal PPAR*α* and PPAR*δ* expression than in the NC group was observed (Figure 3(c)). In our study, the expression of other energy and nutrient sensors (AMPK*α*1/2 and SIRT3) in the liver, small intestine, adipose tissue, and kidney was not different between the HFD-group and the NC-group (Figures 3(d)–3(f)).

A similar decreasing trend of PPAR*α*, PPAR*δ*, and SIRT1 expression in the liver and kidney was observed in HFD mice with steatohepatitis and CKD (Figures 3(c), 3(e), 3(f), and 4(a)). Nonetheless, the expression of SIRT1 in the small intestine and adipose tissue was not different between the NC group and the HFD group. Remarkably, simultaneous activation of hepatic and renal PPAR*α* and PPAR*δ* by preventive or therapeutic elafibranor treatment restored hepatic and renal SIRT1 expression in the HFD group. Elafibranor-related decrease in severity of steatohepatitis was accompanied by an improvement of CKD (Figures 1(c), 3(b), 5(a), and 5(b)). In the electron microscopic images of renal section, a decrease in double membrane structure and autophagosome was observed in the proximal renal tubule of kidney of HFD mice compared to the NC group, which was increased after elafibranor treatment (Figures 4(b) and 4(c)).

### 3.4. Elafibranor Improves Inflammatory and Apoptotic Profiles in HFD Mice with Steatohepatitis and CKD

In HFD mice, the high serum/renal TNF*α* levels, serum caspase 3/7, renal Tunnel stain-assessed apoptotic activity, serum IL-6, serum AST, and ALT were significantly suppressed by elafibranor treatment, and the effect was reversed by EX-527 (Table 2 and Figures 5(f) and 5(g)). Nonetheless, renal IL-6 levels were not different between HFD mice with and without elafibranor treatment.

### 3.5. Renal SIRT1 Activation by Elafibranor Is Accompanied by Normalization of Renal Barrier Markers in HFD Mice

**Figure 6:**
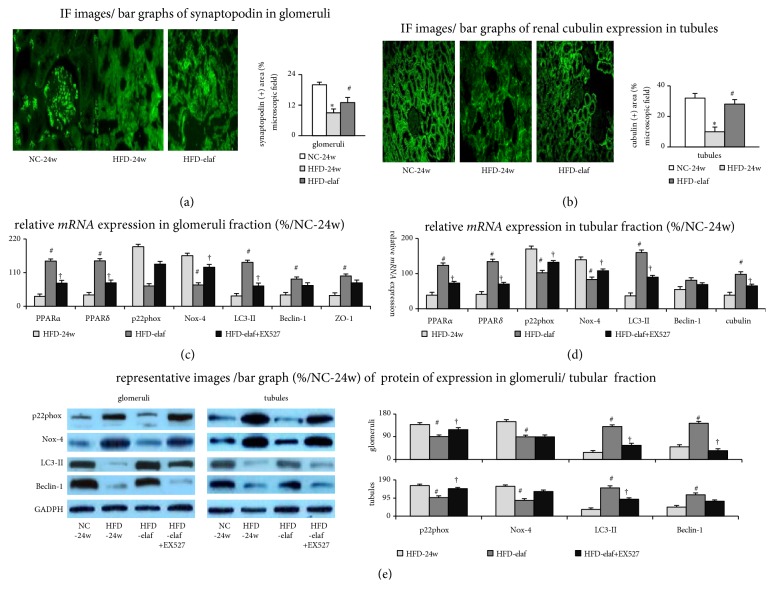
*The suppression of renal oxidative stress was accompanied by the correction of autophagy impairment in HFD-induced CKD mice receiving chronic elafibranor treatment*. For the quantitative evaluation, eight stained tissue sections slides and ten randomly taken pictures were included from each studied group. Representative IF images (200x)/bar graphs of glomerular synaptopodin (a) and renal tubules cubulin (b) expression. mRNAs expression in glomeruli (c) and renal tubules (d) fractions; (e) representative images/bar graphs of western blot for renal proteins expressions in glomeruli and tubular fractions. *∗p*<0.05 versus NC-group; ^#^p<0.05 versus HFD-group; ^†^p<0.05 versus HFD-elaf group.

Decreased renal p-SIRT1 expressions were accompanied by the reduction of the expression of glomerular [synaptopodin (marker of podocyte)/ZO-1] and tubular [cubulin] barrier markers in the HFD-group (Figures 4(a), and 6(a)–6(d)).

Notably, chronic elafibranor treatment partially restored the aforementioned renal barrier markers expression in HFD group (Figures 4(a), and 6(a)–6(d)). In the HFD group, the lower renal PPAR*α*/*δ* expression was associated with less autophagy (Beclin-1 and LC3-II)/barrier (synaptopodin, cubulin, and ZO-1) markers, and more oxidative-stress (p22phox and Nox-4) markers relative to the NC group (Table 2, Figures 3(c), 4(b), and 6).

### 3.6. Elafibranor Treatment Inhibits the Progression of CKD in HFD Mice with Steatohepatitis

In comparison with HFD-24w mice, decreased albuminuria (Figure 5(a)) and improved GFR (Figure 5(c)), reduced tubular injury and tubulointerstitial fibrosis scores (Figure 5(g)), were associated with the restoration of renal PPAR*α*/PPAR*δ*/SIRT1/autophagy (increased Beclin-1/LC3-II) and barrier (synaptopodin/ZO-1/cubulin) markers (Figures 3(c)–3(f), 4(a), and 6), the suppression of renal oxidative stress [p22phox and Nox-4, MPO activity and urinary 8-isoprostane excretion] (Figures 5(e) and 6(c)–6(e) and Table 2), and reduction of renal apoptosis in HFD-elaf mice (Table 2, Figures 5(e), 5(f), and 6(c)–6(e)); these effects were inhibited by concomitant EX527 (SIRT1 inhibitor) treatment. These results suggest that SIRT1 mediated the renal protective effects of chronic elafibranor treatment in HFD mice by activation of PPAR*α* and PPAR*δ* (Figures 4(a), 6(c), and 6(d)).

### 3.7. In Vitro Effects of Elafibranor in HFD-Sera-Pretreated Podocyte/HK-2 Cells

**Figure 7:**
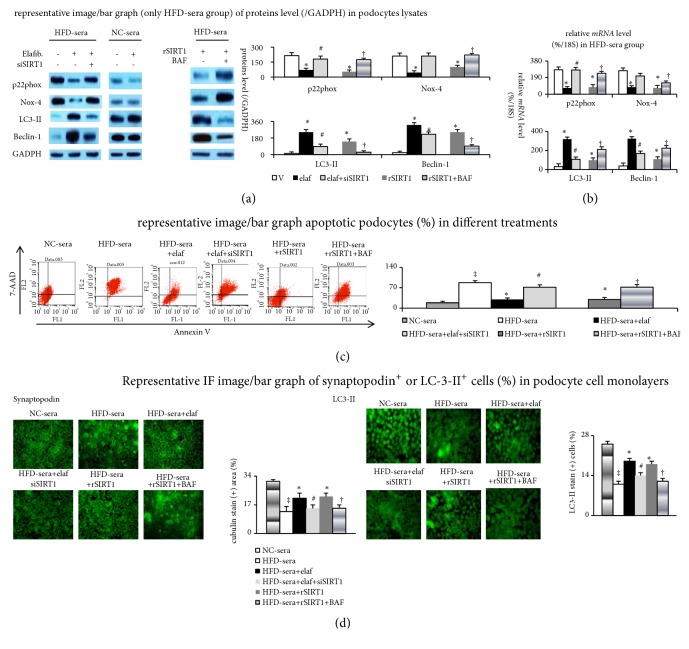
*SIRT1-activated and autophagy-mediated effects of elafibranor on 10% HFD-sera-pretreated oxidative stress and apoptosis in podocytes*. (a) Proteins/(b)* mRNA* levels in lysates of podocytes; (c) flow cytometry-assessed apoptotic cells; (d) IF image/bar graph of synaptopodin/LC3-II expression in podocytes monolayer cells. The optical section data for % of synaptopodin/cubulin/LC3-II (+) area on each slide were evaluated. ‡p<0.05 versus NC-sera group; *∗p*<0.05 versus V-group; ^#^p<0.05 versus elaf-group; ^†^p<0.05 versus rSIRT1-group.

In HFD-sera-pretreated podocytes, compared with NC-sera-stimulated cells, less anti-inflammatory (SIRT1) activity, decreased autophagy (low Beclin-1/LC3-II and autophagy flux index) level, downregulated barrier protein (synaptopodin/ZO-1) expression, impaired albumin endocytosis, and increased oxidative stress (8-isoprotane, p22phox and Nox-4) and apoptosis (caspase 3/7 activity) were noted. These effects were reversed by elafibranor administration (Table 3, Figure 7).

The beneficial effects of elafibranor were eliminated following siRNA targeting SIRT1. Interestingly, recombinant SIRT1 (rSIRT1) has similar effects (anti-inflammation, antiapoptosis, and autophagy activation) as elafibranor. Particularly, both elafibranor+siSIRT1 and rSIRT1-related effects can be blocked by Bafilomycin (blocker of autophagy flux) con-incubation. These results indicate that SIRT1-autophagy cascade plays a pivotal role in elafibranor-related effects of podocytes.

**Figure 8:**
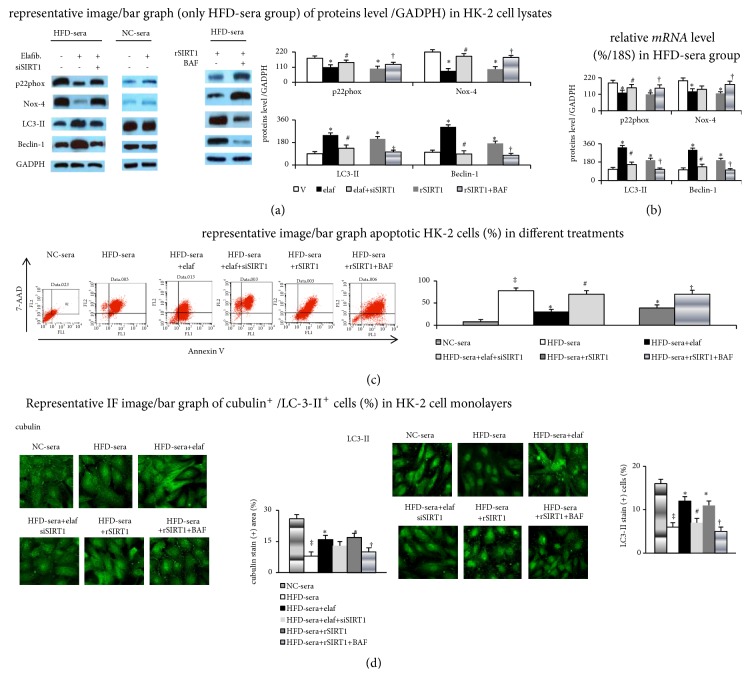
*SIRT1-activated and autophagy-mediated effects of elafibranor on *10%* HFD-sera-increased oxidative stress and apoptosis in HK-2 cells*. (a) Proteins/(b) mRNA levels in HK-2 cell lysates; (c) flow cytometry-assessed apoptotic cells; (d) IF image/bar graph of cubulin/LC3-II expression in HK-2 monolayer cells. ‡p<0.05 versus NC-sera group; *∗p*<0.05 versus V-group; ^#^p<0.05 versus elaf-group; ^†^p<0.05 versus rSIRT1 group.

In cultured HK-2 cells monolayer, the effects of abovementioned treatment were similar to those in cultured podocytes. In particular, the changes in the renal tubular barrier marker, cubulin, were similar as changes in glomerular barrier markers (synaptopodin/ZO-1) in podocytes (Table 3 and Figure 8).

## 4. Discussion

Chronic elafibranor treatment inhibits the progression of HFD-induced CKD in mice in this study. Reduction in GFR and increasing albuminuria are initial markers for the detection of the progression to ESRD [22]. In this study, the renoprotective effects of chronic elafibranor treatment, including preserved GFR and decreased albuminuria, were observed in HFD-induced obese mice with steatohepatitis.

In PPAR*α* knockout and diabetic mice, heavy albuminuria is associated with significant renal inflammation, apoptosis, and fibrosis [13, 23]. In renal tubular cells, PPAR*α* activation protects cells from gentamicin-induced oxidative stress and apoptosis [24]. Both PPAR*α* and PPAR*δ* are highly expressed in kidney [11, 13, 24]. Activation of PPAR*δ* ameliorates tubulointerstitial inflammation in mice with proteinuric kidney disease [25]. PPAR*δ* activation protects cardiomyoblasts from oxidative stress-induced apoptosis [26]. Accordingly, it is reasonable that elafibranor, through renal PPAR*α* and PPAR*δ* activation, improves CKD through inhibition of renal oxidative stress, inflammation, fibrosis, and apoptosis in HFD-induced obese mice with steatohepatitis in our study.

In fact, the renoprotective effects of 12 weeks of PPAR*α* agonist treatment had been reported in HFD-fed obese mice with CKD [27]. In our study, the effects and mechanisms of renoprotective effects of 12 weeks of elafibranor treatment were explored in HFD-fed NASH mice with CKD. Nonetheless, it is mandatory to explore the shortest period of renoprotective effects of elafibranor which need to be evaluated in future studies.

Antiapoptosis and antioxidative stress effects of SIRT1 are accompanied by its anti-inflammatory effects in animals with CKD and steatohepatitis [3, 5, 7, 14–19, 21, 28]. Hepatic and renal SIRT1 were reduced in animals with steatohepatitis and CKD [14, 15]. In our study, concomitant SIRT1 inhibitor (EX527) treatment reversed elafibranor-related benefits, indicating that these effects are SIRT1-dependent. Primarily, antioxidative stress effect is responsible for the activation of renal SIRT1 in HFD-fed mice treated with elafibranor.

By increasing peroxisome function, SIRT1 activation, which is reciprocally stimulated by upregulated PPAR*α* and PPAR*δ*, can prevent the drug-induced renal cell apoptosis and acute kidney injury in mice [29–33]. Decreased SIRT1 expression on podocyte increases cell apoptosis and albuminuria in mice [16, 32]. In our study, siSIRT1 coincubation eliminates PPAR*α*/*δ* agonist elafibranor-related suppression of HFD-sera-induced apoptosis. Meanwhile, rSIRT1 coincubation has similar effects as elafibranor on the reversal of HFD-sera-induced apoptosis. So, in current study, the renoprotective effect of the PPAR*α*/*δ* agonist elafibranor, at least partly, is attributable to SIRT1-mediated inhibition of HFD-induced circulating factors on renal cells and the kidney.

Autophagy promotes cell survival by elimination of damage organelles, which is initiated by increased Beclin-1/LC3-II levels, resulting in increased autophagic flux. Suppression of autophagy (reduced Beclin-1/LC3-II) and autophagy flux induce cell apoptosis. PPAR*α* activation protects the liver from acute inflammation and failure by activating autophagy [33]. PPAR*δ* activation protects human cardiac cells from ER stress-induced injury by stimulating autophagy [34]. Inhibition SIRT1 exacerbates oxidative stress-suppressed autophagy in stem cells [35]. Downregulation of SIRT1 signals is involved in the HFD-induced renal dysfunction in mice [36]. SIRT1 activator suppresses hyperglycemia-induced apoptosis of podocytes via autophagy activation in diabetic mice with nephropathy [37]. In* in vitro* experiments, we revealed that the coincubation with autophagy flux blocker (BAF) abolished elafibranor-related SIRT1-mediated inhibition of NASH-sera-induced pathogenic signals in podocytes and HK-2 cells.

Impaired glomerular protein endocytosis and reduced tubular reuptake of leakage protein can lead to albuminuria. Podocytes are epithelial cells of the outer membrane of renal glomeruli that maintain its integrity. Podocytes damage not only impairs glomerular barrier but also collapses its architecture and leads to advanced renal injury and albuminuria. Restoration of glomerular barrier protein expressions including ZO-1 and synaptopodin (marker of podocyte) avoids albuminuria in diabetic mice [38]. Proximal tubule cells have a capacity to uptake glomeruli-leaked albumin and prevent final leakage. In proximal renal tubules, cubulin mediates the reuptake of leakage albumin from glomeruli to avoid tubulointerstitial inflammation/fibrosis [39]. Oxidative stress-related downregulation of barrier markers worsens albuminuria and tubulointerstitial inflammation/fibrosis [40, 41]. Our study revealed that CKD-related oxidative stress and albuminuria were associated with the downregulation of renal barrier markers in HFD-induced obese mice with steatohepatitis.

**Figure 9:**
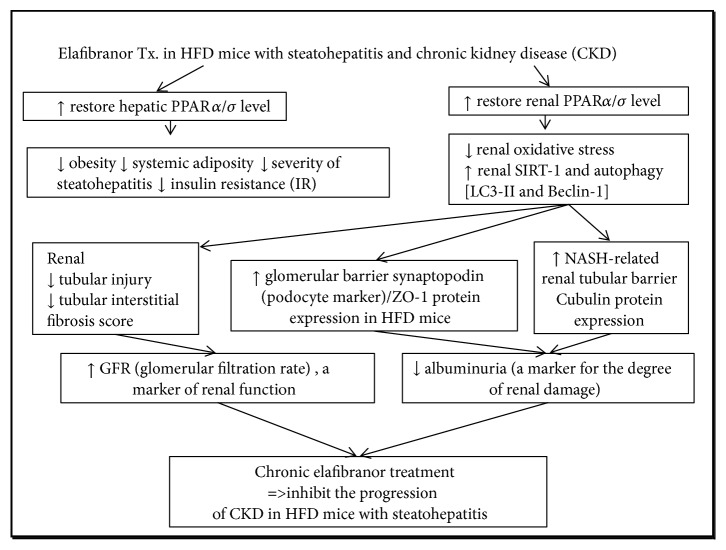
Schematic representative hypothesis for the mechanisms of the inhibition of the progressive of chronic kidney disease (CKD) by chronic elafibranor treatment on HFD mice with steatohepatitis in our study.

In summary, through systemic* in vivo* and* in vitro* approaches, our study revealed that kidney-specific protective effects of elafibranor are attributable to the preservation of glomerular/tubular barrier protein, maintenance of structure, antioxidative stress, and antiapoptosis effects via activation of SIRT-autophagy-mediated protective signals (Figure 9).

This study suggested that elafibranor and strategies aimed at activating SIRT-autophagy are promise for treating high fat consumption which induces steatohepatitis and CKD.

## Figures and Tables

**Table 1:** Primer of various genes measured in this study.

Name of gene	Sequence of sense primer (5-3)	Sequence of anti-sense primer (3-5)
PPAR*α*	5-ATGCCAGTACTGCCGTTTTC-3	5-TTGCCCAGAGATTTGAGGTC-3

PPAR*δ*	5-CCCTTCATCATCCACGACATT-3	5- TGGACTGGCAGCGGTAGAAC -3

Sirt1	5-GCAACAGCATCTTGCCGAT-3	5- GTGCTACTGGTCTCACTT -3

Sirt3	5-CAGCAACCTTCAGCAGTA-3	5-CCGTGCATGTAGCTGTTA-3

AMPK*α*1	5-CAGGGACTGCTACTCCACAGAGA-3	5-CCTTGAGCCTCAGCATCTGAA-3

AMPK*α*2	5-CAACTGCAGAGAGCCATTCACTT-3	5-GGTGAAACTGAAGACAATGTGCTT-3

Beclin-1	5- AATCTAAGGAGTTGCCGTTATAC-3	5-CCAGTGTCTTCAATCTTGCC-3

LC3-II	5- GATGTCCGACTTATTCGAGAGC-3	5- TTGAGCTGTAAGCGCCTTCTA-3

NADPH oxidase subunits p22phox	5- GCGGTGTGGACAGAAGTACC -3	5- CTTGGGTTTAGGCTCAATGG -3

Nox-4	5-ACAGTCCTGGCTTACCTTCG -3	5-TTCTGGGATCCTCATTCTGG-3

ZO-1	5-CCACCTCTGTCCAGCTCTTC-3	5-CACCGGAGTGATGGTTTTCT-3

Cubulin	5- GCTCAACCTCCATTCAATCATA-3	5-GTGCAATCTGTGCTGCTT-3

18S	5- GTAACCCGTTGAACCCCATT-3	5-CCATCCAATCGGTAGTAGCG-3

**Table 2:** Effect of chronic elafibranor (elaf) treatment on the inflammatory profiles of HFD mice with steatohepatitis and CKD.

	NC-24w	HFD-24w	HFD-elaf
Kidney weight (mg)	356 ± 10	423 ± 9*∗*	318 ± 34
[triglyceride, TG, mg/dL]	57 ± 3.9	299 ± 28*∗*	211 ± 14^#^
Hepatic TG levels (mg/g)	98 ± 7	240 ± 18*∗*	200 ± 10^#^
[fasting glucose] (mg/dL)	116 ± 13	243 ± 28*∗*	203 ± 9^#^
[fasting Insulin] (ng/mL)	1.9 ± 0.2	6.9 ± 0.85*∗*	5.2 ± 0.8
Homeostasis model assessment-insulin-resistance (HOMA-IR) index	3.8 ± 0.4	28.8 ± 6.4*∗*	18.1 ± 1.1^#^
[Aspartate aminotransferase] (AST, U/L)	40.9 ± 1.6	117.3 ± 20.1*∗*	79.5 ± 4.3^#^
[Alanine aminotransferase] (ALT, U/L)	46.8 ± 10.3	183.2 ± 6.8*∗*	104.2 ± 13.7^#^
[IL-6, pg/mL]	144 ± 18	223 ± 35*∗*	168 ± 9^#^
Kidney IL-6 (pg/mg protein)	1.8 ± 0.4	7 ± 0.8*∗*	6.2 ± 1.9
[TNF*α*, pg/mL]	12 ± 5	40 ± 11*∗*	29 ± 8^##^
Renal TNF*α* (pg/mg protein)	4 ± 0.8	16 ± 2.3*∗*	9 ± 1.1^#^
Renal MPO activity (U/g)	8.6 ± 1.5	51 ± 20*∗*	31 ± 4^#^
Renal caspase-3 activity (pmol/*µ*g protein)	35 ± 1	90 ± 5*∗*	72 ± 3^#^

NC-24w/HFD-24w: mice receiving 24-week high-fat diet (HFD) or normal chow (NC) feeding and vehicle treatment; NC-elaf/HFD-elaf: HFD- or NC-fed mice receiving 12-week elafibranor treatment from 13^th^ to 24^th^ week of either HFD or NC feeding; HOMA-IR: calculated as ([fasting glucose]×[fasting insulin])/58.32. *∗*, *∗∗p*<0.05,0.01 *vs*. NC-group; ^#^,^##^p<0.05, 0.01 *vs*. HFD-group.

**Table 3:** * In vitro* effects of elafibranor in HFD-sera- or NC-sera-pretreated cultured podocytes/HK-2 monolayer.

Cultured podocytes monolayer	NC-sera	HFD-sera	HFD-sera+elaf	HFD-sera+elaf+siSITR1	HFD-sera+rSITR1	HFD-sera+rSITR1+BAF
SIRT1activity in (fold/buffer group) cell lysates	5.5 ± 0.31	0.44 ± 0.01^†^	3.3 ± 0.06*∗*	1.1 ± 0.02^#^	3.1 ± 0.8*∗*	1.3 ± 0.04^#^
8-isoproatne (pg/mL) level in cell supernatant	462 ± 14	900 ± 5^†^	484 ± 18*∗*	844 ± 10^#^	517 ± 21*∗*	770 ± 11^#^
autophagy flux index in cell supernatant	5.3 ± 0.2	1.5 ± 0.08^†^	4.5 ± 0.5*∗*	2.1 ± 0.3^#^	4.2 ± 0.4*∗*	2.3 ± 0.04^#^
caspase 3/7 activity in cell supernatant (RU)	3 ± 0.08	9 ± 0.18^†^	4.4 ± 0.06*∗*	6.6 ± 0.4^#^	5 ± 0.6*∗*	7.5 ± 0.4^#^
Concentration of FITC-alb in cell lysates	99 ± 22	44 ± 2^†^	77 ± 3*∗*	58 ± 2^#^	71 ± 8*∗*	52 ± 5^#^

Cultured HK-2 cells monolayer	NC-sera	HFD-sera	HFD-sera+elaf	HFD-sera+elaf+siSITR1	HFD-sera+rSITR1	HFD-sera+rSITR1+BAF

SIRT1activity in (fold/buffer group) cell lysates	5 ± 0.28	0.4 ± 0.02^†^	3 ± 0.04*∗*	1 ± 0.04^#^	2.8 ± 0.7*∗*	1.2 ± 0.03^#^
8-isoproatne (pg/mL) level in cell supernatant	420 ± 19	820 ± 4.5^†^	440 ± 22*∗*	650 ± 42^#^	480 ± 25*∗*	700 ± 16^#^
autophagy flux index in cell supernatant	4.8 ± 0.3	1.4 ± 0.07^†^	4.1 ± 0.3*∗*	1.9 ± 0.2^#^	3.8 ± 0.6*∗*	2.1 ± 0.05^#^
caspase 3/7 activity in cell supernatant (RU)	2 ± 0.06	8 ± 0.13^†^	4 ± 0.05*∗*	6 ± 0.34^#^	4.8 ± 0.7*∗*	6.9 ± 0.7^#^
Concentration of FITC-alb in cell lysates	82 ± 17	40 ± 3^†^	70 ± 2*∗*	52 ± 3^#^	65 ± 10*∗*	49 ± 8^#^

Data were showed as mean ± SD; HFD-sera/HFD-sera+elaf/HFD-sera+elaf+siSIRT1/HFD-sera+rSIRT1, HFD-sera+rSIRT1+BAF: HFD-sera-pretreated cells coincubated with vehicle, elaf+siSIRT1, rSIRT1, rSIRT1+BAF in HFD-sera-pretreated group; SIRT1 activity of buffer-group was assigned as 1; RU: relative light unit; autophagy flux index was calculated by the formula [autophagy flux index=LC3-II (indicator of autophagosomes formation) expression levels with Bafilomycin A1 (100ng/mL)/LC3-II expression levels without Bafilomycin-A1]. LC3-II expression was normalized by its GADPH expression level. Concentration of FITC-alb (higher value indicated better endocytosis/reuptake of albumin by podocytes/HK-2) in cell lysates;*∗p*<0.05 *vs*. vehicle-group; ^#^p<0.05, 0.01 *vs.* elaf- or rSIRT1 group; ^†^p<0.05 *vs.* NC-sera group.

## Data Availability

All data supporting the results reported in the article can be found in Division of General Medicine, Department of Medicine, Taipei Veterans General Hospital, Taipei, Taiwan, and can be seen after asking the corresponding author.

## Supplementary Materials

Supplementary Materials Among different groups of male C57BL/6 mice, the whole study includes measurements of serum and tissue metabolic and inflammatory profiles, microscopic analysis of renal tissue for severity of NASH, apoptosis, autophagy and cubulin/synaptopodin, protein and mRNA expressions of various barrier and autophagic markers. Additionally, the roles of SIRT1-autophagy on elafibranor-related effects were evaluated in HFD-sera-pretreated podocytes and HK-2 cells system. The additional detail materials and methods are included in supplementary materials and methods.
