# Supplementary material for: Elafibranor Inhibits Chronic Kidney Disease Progression in NASH Mice
Source: Biomed Res Int. 2019 Jun 19;2019:6740616. doi: 10.1155/2019/6740616 (PMC6607733; doi:10.1155/2019/6740616)
Supplement: Supplementary Materials — Among different groups of male C57BL/6 mice, the whole study includes measurements of serum and tissue metabolic and inflammatory profiles, microscopic analysis of renal tissue for severity of NASH, apoptosis, autophagy and cubulin/synaptopodin, protein and mRNA expressions of various barrier and autophagic markers. Additionally, the roles of SIRT1-autophagy on elafibranor-related effects were evaluated in HFD-sera-pretreated podocytes and HK-2 cells system. The additional detail materials and methods are included in supplementary materials and methods. [file 6740616.f1.pdf]

Supplement materials and methods

Animals. This study was approved by the Animal Experiments Committee of Yang-Ming University and was performed according to the “Guide for the care and use of laboratory animals” prepared by the National Academy of Science, USA and the ARRIVE guidelines. At the end of the experiments, the rats were euthanized with 2-3 times the anesthetic dose of zoletil. All efforts were made to minimize animal numbers necessary to produce reliable results and suffering was reduced by administering anesthetics (zoletil and xylocaine).

Male C57BL/6 mice (Jackson Laboratories, Bar Harbor, ME), 8 week old were housed in temperature- and humidity-controlled rooms, kept on 12 h light/dark cycle, and provided unrestricted amounts of food and water. High-fat-diet (HFD, D12492) used in this study contains 34.9% fat (mostly saturated) by weight and yield 60% of calories from fat (caloric density 5.24 kcal/g). Elafibranor (2-[2,6 dimethyl-4-[3-[4-(methylthio)phenyl]-3-oxo-1(E)-propenyl]phenoxyl]-2-methylpropa noic acid) were purchased from Genfit.

Serum and tissue metabolic and inflammatory profiles. Serum biochemistry data were measured using a standard auto SMAC analyzer (Roche Diagnostics Gmbh, ANNHEIM, Germany). Serum insulin, TNFα, and IL-6 levels as well as caspase-3/7 activity (relative light unit, RU) were measured using ELISA and colorimetric assay kit (Biovision, San Francisco Bay Area, CA, USA). Triglyceride content was measured by a triglyceride Colorimetric Assay Kit (Cayman Chemical Company, Ann Arbor, MI, USA). Renal IL-6/TNF-α levels, myeloperoxidase (MPO) and caspase 3 activities were measured by ELISA kits [BD Biosciences (San Jose, CA). Hepatic and renal SIRT1 activities [relative fluorescence units (RFU)] were determined with a SIRT1 fluorometric Kit (Abcam).

Histologic analysis. Nonalcoholic fatty liver disease activity score (NAS) were measured by H-E-stained liver section. In the paraffin-embedded tissue, the whole kidney cortex (ten random pictures/per section) of the H-E and periodic acid-Schiff (PAS)-stained renal section was examined independently by two investigators. The whole kidney cortex (ten random pictures/per section) was evaluated to score the renal tubular damages and tubulointerstitial fibrosis.

With an ApopTag Peroxidase *In Situ* Apoptosis Detection Kit (Chemicon, CA, USA), in Terminal deoxynucleotidyl transferase-mediated dUTP nick end labeling (TUNEL)-stained renal cortex section (×200), TUNEL-positive glomerular and tubules cells undergoing apoptosis were calculated. At least 10 glomeruli and 4-6 non-overlapping random tubular fields per biopsy were used to determined apoptosis, which is expressed as the mean number ± SD per field.

Renal electron microscopic and immunofluorescence analysis. For electron microscopy, renal samples were fixed with a 2.5% glutaraldehyde solution buffered at pH 7.4 with 0.1M of Millonigs phosphate at 4℃ for 2 hours and post-fixed with a 1% osmium tetroxide solution at 4℃ for 1 hour. After dehydration in graded concentrations of ethanol, samples were embedded in Nissin EM Quetol 812 epoxy resin. Ultrathin section measuring 80nm thick were cut with an ultramicrotome. Sections were stained with uranyl acetate and lead citrate and observed with a Hitachi H-7650 electron microscope (Hitachi High-Technologies Corporation, Tokyo, Japan) at 80kV. The isolated membranes and autophagosomes on the ultrathin section in proximal renal tubule of kidney were calculated by electron microscopy at 1,200x magnification. Meanwhile, each slide was evaluated for the numbers of cubulin/synaptopodin (+) cells per $1mm^2$ in the FITC images. For the quantitative evaluation, eight stained tissue sections slides and ten randomly taken pictures were included from each studied group.

Protein and mRNA measurements. In addition to the podocytes/HK-2 cell lysates, glomerular and tubular fractions were separated by graded sieving from the renal homogenates. Then, various proteins and mRNAs were measured with antibodies, including SIRT1, phosphorylated-SIRT1, LC3-II, Beclin-1, cubulin, ZO-1, p22phox, Nox-4 and GADPH, purchased from R&D Systems (Inc., Minneapolis, USA), Abcam (Cambridge, MA, USA) or Santa Cruz (Biotechnology, CA, USA) and various primers (supplement Table 1). Mouse podocytes and HK-2 cells were purchased from the CELPROGEN (3914 DEL, AMOBLVD, SUITE901, TORRANE, CA90503) and Bioresource Collection and Research Center (BCRC, Hsin-Chu, Taiwan).

For gene expression measurements, total *RNA* was isolated from frozen liver, small intestine, adipose tissue, and kidney tissues by Trizol reagent as indicated by the manufacturer’s instructions (Invitrogen, Carlsbad, CA, USA). The quality and integrity of the *RNA* were confirmed by agarose gel electrophoresis and ethidium bromide staining, followed by visual examination under UV light. Reverse transcription was performed using the MMLV reverse transcriptase 1st-strand cDNA Synthesis Kit (EPICENTRE, Madison, WI, U.S.A.). The reverse transcriptase reaction was performed with 200 ng *RNA* in a final volume of 20 μl. The thermocycler conditions consisted of a first step at 25℃ for 10 min, followed by a second step at 37℃ for 120 min. The samples were then heated at 85℃ for 5 min and cooled to 4°C. The cDNA was stored at −20°C until further analysis. Then, real-time quantitative PCR was performed on an Applied Biosystems 7900HT Sequence Detection System (Applied Biosystems, Foster City, CA, USA) using specific primers (Table 1) and SYBR Green probe. Each sample was assayed in triplicate with 30 ng of

input cDNA per well in a volume of 25 μl reaction containing QuantiTect SYBR Green PCR Master Mix and specific primers. The cycling conditions were two holding stages, the first at 50℃ for 20 sec and the second at 95℃ for 10 min, followed by 40 cycles at 95℃ for 15 sec and at 55℃ (Beclin-1, ZO-1 and cubulin), at 57℃ (AMPKα1, AMPKα2, PPARα, PPARδ and 18S) or at 60℃ (p22phx, NOX4, and LC3II) for 30 sec, and a Melt Curve stage at 95℃ for 15 sec, 60℃ for 1 min, 95℃ for 30 sec and 60℃ for 15 sec. The Melt Curve analyses were performed to confirm the identity and purity of the amplified products. Each reaction was performed twice in order to ensure technical reproducibly of the assays. Each pair of primers was tested in independent plates together with all the samples. A negative control (no template control) was included in each plate in order to test for general contamination. The cycle number at which the reaction crossed an arbitrarily-placed threshold (Cycle threshold values, C_T) was determined for each gene and the relative amount of each miRNA to 18S r*RNA* was described using the equation $2^{-\Delta C}{}_T$ where $\Delta C_T = (C_{TmRNA} - C_{T18S\ rRNA})$. Relative *mRNA* levels were determined using a standard curve generated by pool of *RNA* samples and normalized to 18S r*RNA*.

Roles of SIRT1-autophagy on Elafibranor-related effects on HFD-sera-pretreated podocytes and HK-2 cells. HFD/NC-sera were obtained from NC-24w and HFD-24w mice. Briefly, 1mL of whole blood via tail vein was collected, allowed to clot and centrifuged for 20min at 2500rpm. Serum was removed from the centrifuged samples and stored frozen until used. All serum utilized was thawed and heat inactivated 56℃ prior to use in cell culture experiments.

To mimic the impacts of circulating factors of HFD mice on abnormalities of renal microenvironment, various measurements were undertaken in 10% HFD-sera-pretreated podocytes/HK-2 cells. After 24 hours of starvation with serum free DMEM, the cultured HK-2/podocytes grown in the presence of 10% HFD/NC-sera for another 24 hours. Significantly, 10% HFD-sera incubation suppressed the SIRT1 activity in cell lysates of podocytes/HK-2 cells. A preliminary dose-finding experiment revealed that, among different concentrations (5, 10, 15, 30μM) of elafibranor, maximal stimulation of SIRT1 activity on HFD-sera-pretreated cells was noted at 15μM of elafibranor. Meanwhile, siSIRT1 was transfected into cells and maximal blockade of elafibranor-activated SIRT1 activity was noted at 100μM. Control siRNA (nontargeting siRNA; Santa Cruz) served as control. For siRNA-mediated gene knockdown, siSIRT1 or control siRNA (nontargeting siRNA; Santa Cruz) were transfected into cells. The sequences of siSIRT1 were 5′-ACUUUGCUGUAACCCUGUA-3′. siRNAs were transfected into HK-2 cells/podocytes with Lipofectamine™ RNAiMax according to the manufacturer's

protocol.

Meanwhile, the role of SIRT1 in elafibranor-mediated effects was validated using rSIRT1. We found that rSIRT1 (300μM) had similar effects as elafibranor (15μM) to reverse HFD-sera-suppressed SIRT1 activity in cells. To examine the SIRT1-mediated effects of elafibranor on autophagy, HFD-sera-pretreated cells were incubated with bafilomycin A1 (BAF, 100ng/mL, a blocker of autophage flux) concomitantly with elafibranor (15μM) or rSIRT1 (300μM). The *in vitro* experiments revealed that 0.1% DMSO [solvent for elafibranor], siSIRT1, recombinant SIRT1 (rSIRT1, Sigma-Aldrich) and bafilomycin A1 (BAF, Sigma-Aldrich)] and scrambled siRNA did not affect the cell viability. For the following *in vitro* experiments, vehicle (V), elaf, elaf+siSIRT1, rSIRT1, rSIRT1+BAF group in either HFD-sera-or NC-sera-pre-treated cells were included.
